# Quick Spreading of Populations of an Exotic Firefly throughout Spain and Their Recent Arrival in the French Pyrenees

**DOI:** 10.3390/insects13020148

**Published:** 2022-01-29

**Authors:** Marcel Koken, José Ramón Guzmán-Álvarez, Diego Gil-Tapetado, Miguel Angel Romo Bedate, Geneviève Laurent, Lucas Ezequiel Rubio, Segimon Rovira Comas, Nicole Wolffler, Fabien Verfaillie, Raphaël De Cock

**Affiliations:** 1 LABOCEA (R&D Unit)–CNRS, 120 Avenue Alexis de Rochon, 29280 Plouzané, France; nicole.wolffer@orange.fr; 2 Independent Researcher, 41008 Sevilla, Spain; gusanosdeluz@gmail.com; 3 Department of Biodiversity Ecology and Evolution, Universidad Complutense de Madrid, 28040 Madrid, Spain; diego.gil@ucm.es; 4 Independent Researcher, Villanueva de la Serena, 06700 Badajoz, Spain; miguel.romo.bedate@gmail.com; 5 OVL Citizen Science Project, Groupe Associatif Estuaire, 85440 Talmont St. Hilaire, France; genevievelaurent66@gmail.com; 6 Faculty of Exact and Natural Sciences, University of Buenos Aires, B1657, Buenos Aires C1428EGA, Argentina; lucasrubio00@hotmail.com; 7 Grup Cucadellum–ICHN Citizen Science Project, Catalan Institution of Natural History, 08001 Barcelona, Spain; srovira@xtec.cat; 8 Groupe Associatif Estuaire, Port de la Guittière, Rue de Louza, 85440 Talmont St. Hilaire, France; capsurlaplanetevie@gmail.com; 9 Evolutionary Ecology Group, University of Antwerp, B-2610 Antwerp, Belgium; rdecock@hotmail.com

**Keywords:** citizen science, invasive, exotic species, phenology, flight behavior, distribution

## Abstract

**Simple Summary:**

Here, we describe the spreading of a South American firefly in Europe that established itself in northeastern Spain, probably in 2016. The population is expanding quickly (by about 10 km per year) and arrived in 2020 in France. The observations were collected through three citizen science platforms, the Spanish Gusanosdeluz, the Catalan Grup Cucadellum and the French “Observatoire des Vers Luisants et des Lucioles”. This underlines the importance of this type of approach involving the general public for academic research and in following this potentially invasive species. In 2018, the species was described as new to science, but we discovered that it was already designated as *Photinus signaticollis* by Emile Blanchard in 1846 and originated from Uruguay and Argentina. Evidence is provided for the identity between Spanish and Argentinian specimens via aedeagus analysis and by culturing an Argentinian (and French) larva to adulthood. Therefore, we propose that the original 1846 name is reattributed. Some of the animal’s biology is described (phenology in Spain/France and Argentina, land use, etc.) and we show the earthworm-eating larvae and the pupal stage. The potentially large consequences of having a foreign firefly species that is theoretically invasive, and that eats earthworms, in a European setting, are discussed.

**Abstract:**

In August 2018, a firefly (*Coleoptera: Lampyridae*) of American origin was observed in several localities in Girona (Catalonia, Spain) and was described as *Photinus immigrans* by Zaragoza-Caballero and Vinolas, 2018. Here, we show that this species dispersed very quickly throughout northeastern Spain and was, in 2020, observed in the French Pyrenees. The animal’s quick progress is documented, and part of its biology is described (dispersion speed, land use, phenology, identification of all life stages). An additional population was localized in Extremadura, and its special status is discussed. We were able to determine its Argentinian–Uruguayan origin and propose, therefore, to consider *Photinus immigrans* as a synonym of *Photinus signaticollis* (Blanchard, 1846) (=*Photinus immigrans* Zaragoza-Caballero and Viñolas, 2018, syn. nov.). Our data clearly show that at least the Catalan and French populations are spreading very quickly and are able to settle permanently if adequate ecosystems are found. The species is highly expansive and may well be invasive; our citizen science platforms are ideally suited to monitor their progress throughout Spain and France. This is important for avoiding future ecological problems with diverse native faunas, such as glow-worms, fireflies and earthworms. If no ways are found to stop the species’ progression, the animals will quite probably invade substantial areas of France, Spain and the rest of Europe in the years to come.

## 1. Introduction

Fireflies, glow-worms or lightning bugs (*Coleoptera, Lampyridae*) are a group of about 2200 described beetle species distributed worldwide [1]. Both larvae and adults of this group are ferocious predators, feeding mainly on terrestrial gastropods or earthworms [2,3,4]. Lampyrids use bioluminescence to attract mates or prey or announce their unpalatability [2,4,5,6]. This light emission renders their detection quite easy and makes them a subject of choice for citizen science.

Citizen science is a powerful and innovative way to tackle large-scale surveys that individual scientists are completely unable to perform, because for them (in contrast to the general public), it is impossible to perform observations “everywhere, any day and at any time of the year”. In France, “l’Observatoire des Vers Luisants et des Lucioles (OVL)” (>15,000 observations/year) and the Spanish “¿Has visto una luciérnaga? (Gusanosdeluz GDL)” (>200 observations/year) have been building an inventory of their countries’ firefly and glow-worm fauna since 2015 and 2009, respectively. These nation-wide citizen science projects both target the general public and use naturalist associations as regional relays. In addition, in 2020, a regional project, “Grup Cucadellum-ICHN (GC)”, was launched to significantly intensify observations in the Catalan area (northeastern Spain).

The primary goal of these three citizen science projects is to obtain a detailed idea of the presence/absence of glow-worms and fireflies in France and Spain. Simple questions are asked to the general public, such as “where”, “when”, “how many” and “which species of glow-worms or fireflies did you see?”. If people answer these first questions, we asked them to dig deeper, send photographs and describe, for instance, the habitats, the presence of artificial light sources, their gardening practices, etc. Data are collected and correlated with natural and human landscape features to understand the animals’ biologies and follow their populations in a nation-wide way, impossible by other means. For most of the West European species, photo identification is a feasible tool, based on easy-to-use field characteristics, without the need to capture specimens.

Our Lampyrid-centered citizen science projects coexist with several general biodiversity photo-sharing platforms (e.g., i-Naturalist, Ecoregistros, Biodiversidad Virtual, Observado.org), on which information and determinations are shared.

Now, the close collaboration between our three Lampyrid projects clearly demonstrates the power of such citizen science approaches, since, for the first time by these means, we were able to highlight the very rapid progress of an expansive (and perhaps invasive) exotic firefly species.

Invasive alien species represent one of the main causes of biodiversity loss [7], and due to globalization and the consequent transport of goods, an increasing number of new species are being introduced into new territories [8,9,10]. Detecting and reporting non-native species are the first steps to be able to implement curative measures and prevent their spread in allochthonous territory. Research and new knowledge about alien species (e.g., their taxonomy, ecology or dispersal capacity) are vital in understanding the possible role of an invasive species in a colonized area.

In August 2018, an exotic firefly, most probably of American origin, was observed in several localities in Girona (Catalonia, Spain) [11]. It was described as *Photinus immigrans* by Zaragoza-Caballero and Viñolas, 2018 [12]. In the course of 2018, 2019 and 2020, the Departament de Territori i Sostenibilitat of the Generalitat de Catalunya and the Lampyrid citizen science platforms GDL and GC received new sightings of this exotic species and, to our surprise, OVL had detected them already in 2020, over the Spanish border in the French Pyrenees

The current work analyzes all the distribution data available for this exotic firefly (provided in Supplementary Materials) from the above-mentioned citizen science platforms. They provide insight into the following: (1) how quickly this species is able to disperse over years by calculating expansion rates; (2) the species’ biology, including phenology and images of larval and pupal stages; (3) a second verified location in central western Spain, in Extremadura, 750 km away from the Catalan populations, and an additional suggestive sighting of the species from La Rioja (central eastern Spain); (4) the species’ Argentinian–Uruguayan origin; (5) by comparing the species descriptions and performing aedaegus analysis and larval cultures, the synonymization of this species under *Photinus signaticollis* (Blanchard, 1846) (=*Photinus immigrans* Zaragoza-Caballero and Vinolas, 2018, syn. nov.) is proposed.

Finally, we discuss the possibility of the species being invasive, and we argue for the urgent need for more research on its biology and continued monitoring of its rapid dispersal, which may lead to a quick invasion of Western and Central Europe and problems with native glow-worm, firefly and earthworm faunas.

## 2. Materials and Methods

### 2.1. Data Collection and Analysis

Currently available records of the *Photinus immigrans/signaticollis* species in Spain, France, Argentina and Uruguay between 2016 and October 2020 were collected from the following firefly-specific citizen science platforms (all accessed on 31 October 2020):

− GusanosdeLuz http://www.gusanosdeluz.com/ (gusanosdeluz@gmail.com (GDL))
− Grup Cucadellum https://cucadellum.cat/ Institució Catalana d’Historia Natural (info@cucadellum.cat) (GC)
− Observatoire des Vers Luisants et des lucioles http://www.asterella.eu/ (OVL)

In addition, records were collected from the following general naturalist citizen science platforms:

− Biodiversidad Virtual www.biodiversidadvirtual.org/
− iNaturalist www.inaturalist.org/
− EcoRegistros www.ecoregistros.org

Some of the data reported in [12] were also included (duplicates were removed, data used are indicated in Table S1, Supplementary Materials).

In 2020, the “DTSG (Departament de Territori i Sostenibilitat)” of the Generalitat de Catalunya released a map with the observation collection of *P. immigrans* for the Catalonian region of Girona between 2016 and 2020, which provided inspiration for the present study concept and analysis.

Prior to analysis, all records were verified for the mention that animals were indeed “flashing AND flying”, images were checked and duplicates were removed, i.e., removal of sightings at the same location and on the same date, several users reporting the same population at the same location and date, or the same photo and data posted on several platforms. The photographs, provided by many citizen scientists, allowed us a species determination without any doubt.

Maps and dispersal analyses were performed in ArcGIS v 10.4.1 for desktop (ESRI, 2015). Dispersal rates were calculated using the Near tool, considering the minimum Euclidean distance between the 2019 and 2020 records (the consecutive years with the highest number of records) and were corrected by a geodesic projection, following [8]. Distances smaller than 1 km were ignored to avoid pseudo-replication among years. Very large distances (more than 17 km) were also ignored under the assumption that such distances may be due to new *P. signaticollis* introduction events through human transport or due to sampling bias, blurring the natural dispersal of the species.

In order to determine the original location of the Spanish/French *Photinus* sp., we tracked data and photographs of fireflies from the Americas on several citizen science platforms (iNaturalist and EcoRegistros) for species resembling *P. signaticollis*/*immigrans*. Once resembling fireflies were found, local citizen scientists were contacted and one of us (L.E.R.) caught the local specimens that, through aedeagus analysis and larval culture, allowed the identification of the *Photinus immigrans* as *Photinus signaticollis* (Blanchard, 1846).

CORINE land coverage:

https://land.copernicus.eu/user-corner/technical-library/corine-land-cover-nomenclature-guidelines/docs/pdf/CLC2018_Nomenclature_illustrated_guide_20190510.pdf (accessed date 20 October 2020).

### 2.2. Field and Photographic Identification Characteristics of Photinus signaticollis

Pronotum coloration is often used as field characteristics for *Photinus* spp. (references in [2,4,6,11,12]).

**Figure 1:**
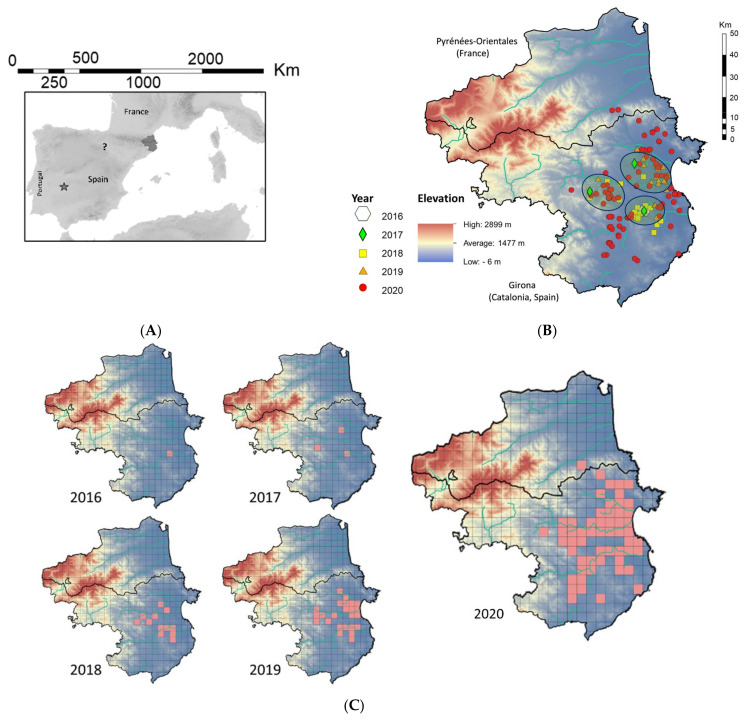
Observations in northeastern (Girona), and central western Spain (Extremadura), as well as in southeastern France. (**A**) Overview of all European observation sites; the asterisk points to the observation in Extremadura and the question mark shows the La Rioja sightings (see below). The observation from north central Spain (La Rioja) needs further verification. (**B**) Visualization of the Girona and French observations. The background colors indicate the elevation (digital terrain model). Light blue lines mark rivers, whereas dark blue ovals show the main focal areas of initial *Photinus* sp. presence. The central black line is the French–Spanish border. (**C**) Time course of observations are shown as presence (pink squares) in 5 × 5 km^2^ squares.

- Morphologically, the adults have a body plan like most *Photinus* species (especially nocturnal and flashing species [2,13] (see Figure 1 within), with lateral pinkish markings (curved thickened lines to oval spots) around the dark brown, almost square, central spot on the pronotum. The pinkish markings are laterally flanked by dark areas, with a similar color to the dark median spot (absent in other nocturnal *Photinus* species, where, usually, pale colors laterally flank the pinkish areas on the pronotum); most commonly, the pink markings show as lateral pink lines, slightly curved towards the center of the pronotum, with a faint thickening in the center on the hollow side of the pink line facing outwards.

This is reminiscent of pronotal colorations seen in diurnal *Photinus* (i.e., previously genus *Ellychnia* [13]), *Lucidota* and *Pyractomena* firefly species, present in the region of origin. This suggests that they are part of an aposematic (Müllerian) mimicry complex, meaning that different species benefit from mimicking each other’s warning color defenses, i.e., to signal toxicity or unpalatability to a shared predator community [14,15,16].

- Greyish-brown color of elytra with clear pale margins and often a more or less apparent (sometimes absent) pale vitta (shoulder stripe) reaching from the shoulder to (almost) the pale margin at the tip of the elytrum. Shoulder vittae are uncommon in other Photinus spp. and are more typically found in Photuris spp.
- Elytra shape often taper (broadest at shoulders) and are sometimes parallel; elytra are not always touching in the middle, often slightly opening towards the apex.

### 2.3. Spanish Collection and Deposit of Specimens

Two *Photinus* specimens were collected at Villanueva de la Serena (Badajoz, Extremadura) by J.R.G.A. in September 2020. Capture date: 15 September 2020. One specimen was used for taxonomic (aedeagus extraction) and genetic analyses. The second Extremaduran specimen was deposited at the Museum of Natural Sciences of Madrid (Collection N° **MNCN_Ent 269418**); two French males and two French females were collected in September 2021 and were also deposited at this institution (**MNCN_Ent 296122**); these were together with two males from Girona, previously collected by M. Casanovas and E. de la Arada in the framework of GDL observations (**MNCN_Ent 255158** and **MNCN_Ent 259490**, [17]).

### 2.4. Aedeagus Preparation

The male “sexual apparatus” (*aedeagus*) was extracted by micro-dissection. After removal of the remaining “muscle tissue” with a several hours incubation in 10% KOH solution, the *aedeagus* was photographed in a 50% glycerol solution under different angles on a Keyence numerical microscope.

### 2.5. Original Description of P. signaticollis by Blanchard

The original description of *P. signaticollis* by Blanchard [18], in Latin, is as follows:

*Elongatus*, *cinerescens*; *antennis nigris*; *prothorace facido*, *macula media nigra*,

*lineola divisa*; *elytris cinereis*, *marginibus pallidioribus*. Long. 14 millim.

Additionally, the description in French is as follows:

Cet insecte est étroit et al.longé; la couleur générale de son corps est d’un gris brunâtre assez clair. Les antennes sont noires.

Le prothorax, aplati sur les côtés, ayant ses bords très-peu relevés, est arrondi en demi-cercle, d’un jaune pâle, avec une tache noire médiane presque carrée, divisée par une ligne enfoncée; de chaque côté de cette tache on distingue une nuance un peu rosée. L’écusson est d’un brun claire.

Les élytres allongés, d’un gris cendré uniforme, ont une bordure claire.

Les pattes sont brunes, avec la base des cuisses et les hanches plus jaunâtres.

L’abdomen est brun, avec les deux anneaux lumineux d’un jaune soufre.

Ce Lampyre a la forme du *L. occidentalis*, Oliv., auprès duquel il doit se placer; les taches de son corselet le distinguent au premier abord de cette espèce. M. d’Orbigny l’a trouvé dans la province de Maldonado, dans le fond des dunes, près du bord de la mer.

The English translation is as follows:

This insect is narrow and elongated; the general color of its body is a fairly light brownish-gray. The antennae are black.

The *prothorax*, flattened on the sides, has its edges very slightly raised, is rounded in a semicircle, of a pale yellow, with a median black spot of an almost square shape, divided by a sunken line; on either side of this spot we can distinguish a slightly pinkish or rosy shade. Light brown *scutellum*.

The elytra elongated, uniformly ash gray **^#^**, have a light border.

The legs are brown, with the base of the thighs and the hips more yellowish.

The abdomen is brown, with the two luminous segments coloured sulfur yellow.

This firefly has the shape of *L. occidentalis*, Oliv. (=*Photinus occidentalis* (Olivier, 1790)), near which it must be placed; the spots of its *pronotum* distinguish it at first sight from this species. Mr. d’Orbigny found it in the province of Maldonado (Uruguay), at the base of the dunes, near the edge of the sea.

**^#^** Please note that, in our own observations, the Argentinian and Franco–Spanish specimens often display, in both male and female specimens, a paler “shoulder strip” (*vitta*) on each elytron.

## 3. Results

### 3.1. Initial Spanish Observations

In August 2018, flashing fireflies were reported in Catalonia (Spain) both by “citizen” and academic scientists [11,12,17]. The discovery was announced to the local authorities (Generalitat de Catalunya, [17]). As in Europe, firefly flashing during flight is something typical for the *Luciola* genus, hope flourished that it may be the firefly *Luciola lusitanica* (Charpentier, 1825), that lives in Portugal, in the French Mediterranean area around Nice, in Italy and possibly in the Balkans [19,20,21].

Spanish citizen scientists had been looking for this species for years, as according to the old literature, it should occur or have occurred in Spain [22,23]. However, images of the observed firefly showed that we were dealing with an exotic genus, completely different from *Luciola*, although with similar bioluminescence behavior. The animal belonged to the large *Photinus* genus (*Coleoptera*, *Lampyridae*, *Photinini*, *Photininae*, *Photinus*, Laporte, 1833) that is only known from the Americas [11]. Soon after, the species, apparently unknown to science, was described as *Photinus immigrans* Zaragoza-Caballero and Viñolas, 2018 [12].

The first observation (registered in 2018) dates back to 2016. Two more occurrences were recorded in 2017, separated by 27.6 and 23.3 km from the first record (Figure 1B). With these three occurrence locations and the separation between them (higher than the mean dispersal distance, see below), we cannot be sure of the location of the original introduction, or how long this exotic firefly has been on European territory.

As can be seen in the time course of Figure 1C, the species had a rather rapid expansion in Catalonia. Note that the dispersal seems to follow the river valleys.

### 3.2. Expansion Rate

**Figure 2:**
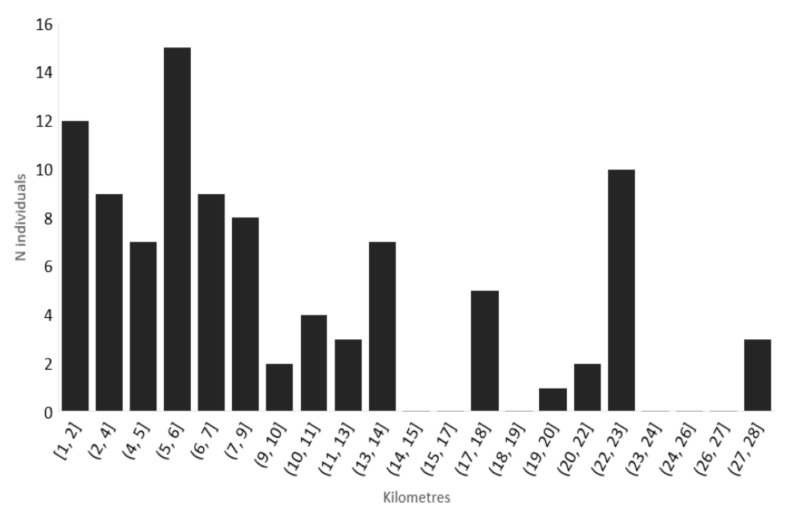
Dispersal of the *Photinus firefly* calculated with the nearest distance between the 2019 and 2020 records. The calculated average dispersal distance of the 2019–2020 range corresponds to 6 km/year with a SD of 3.5.

An approximate expansion rate was calculated over the last two years (Figure 2), as described in the Materials and Methods Section.

### 3.3. French Observations

As the 2020 DTSG data showed that the species was approaching the French border in 2020, one of us (R.D.C.) suggested to check the observations sent to the French “OVL” for “flashing flying fireflies” in the French department “Pyrénées-Orientales (N°66)”, which includes the Perpignan region near the Catalan border. Two localities were identified where flying and flashing insects had been observed from June 2020 (two most northern red dots in Figure 1B). When these sites were prospected, a big *Photinus* population (40–50 blinking specimens/evening) was found in the village Maureillas-las-Illas, whereas in “Les Cluses”, a single flying and flashing specimen had been observed. This particular population exhibited flashing and flying individuals until the 1st day of November. After that date, no flying specimens were observed anymore, but flashing animals remained present on the ground vegetation until the 14 November. So, apparently, in France in 2020, the adult reproduction period extended from mid-June to the beginning of November.

The territories beyond the Pyrenees had been colonized after only 3 or 4 years. The pioneers had to bridge a bird fly distance (air distance) of at least 28 km (https://www.distancefromto.net/ (accessed date 20 October 2020)) between the 2017 most northern populations recorded in the south of the mountain range (Avinyonet de Puigventós, Catalonia, Spain) and the village of Maureillas-las-Illas in France. The simplest and most logical natural path to traverse the Pyrenees in this area is the corridor formed by the rivers Llobregat and Rome, separated by the Perthus Pass (290 m). The first individuals passed the border in early 2020 or maybe the year before. This distance (28 km) could be overcome in one or various steps, because some specimens seem to be able to disperse over distances between 15 and 23 km, as can been seen in Figure 2.

### 3.4. Extremadura Observations

In October 2018, another observation of many flashing and flying fireflies was sent to GusanosdeLuz from Villanueva de la Serena (Badajoz, Extremadura) about 100 km from the Portuguese border (asterisk, B&W map, Figure 1A). Again, *Luciola lusitanica* was expected, as the species is abundant in Portugal, but after receiving photos and upon visits in 2019 and 2020 a small colony of the *Photinus* was found in a single privately owned 3000 m^2^ area. The owner told us that he had witnessed these fireflies’ flash displays on his property for at least 40 years.

Although neighborhood locals were interrogated, no other nearby sites could thus far be identified. Apparently, this small population remains in its distribution area, taking advantage of suitable habitat conditions and seems, thus far, not to disperse beyond it. From an ecological point of view, this absence may be explained by the isolation of the property, characterized by a perennial herb layer structure (*Cynodon dactylon* (L.) Pers., 1805) prairie in a residential area, in contrast with the surrounding sandy soil agriculture fields. Future genetics studies will have to clarify whether there is a genetic relationship with the Girona populations, or whether the populations were newly introduced from their native American grounds.

### 3.5. La Rioja Information

GDL received another observation from Northern Spain (question mark, B&W map, Figure 1A) that pointed to the presence of *Photinus* specimens. Two flying and flashing fireflies were spotted entering the observer’s house (San Roman de Cameros, La Rioja, Spain) at the end of July–beginning of August 2020 at around 10 p.m. Unluckily, the observer did not take photographs, but the species was clearly new to her and never observed before, while the typical glow-worm-type fireflies are locally abundant and well known to her from the region. The description of flying fireflies with flashing abdominal lights, some orange-colored parts and blackish backs, that looked like a “sunflower seed”, matches with the description for *P. signaticollis* (Figure 7, below). Apart from this report, unfortunately no new sightings happened in the summer of 2021 and no visual proof in the form of videos or photos could be collected, so we have to treat this observation with caution.

### 3.6. Synonymisation of Photinus Immigrans and Photinus signaticollis (Blanchard, 1846)

Subsequently, citizen science provided us once again with even more interesting information. The iNaturalist and EcoRegistros websites permit searching images of American *Photinus* species (a detailed search strategy is provided in Supplementary Data S1). A *Photinus* species living in Argentina and Uruguay seemed, at first sight, identical to the Spanish “*P. immigrans*”, although the identification to the species level was lacking in all these records.

Checking the original species descriptions for the Argentinian–Uruguayan region [18,24], one particular *Photinus* species, named *Photinus signaticollis* (Blanchard, 1846), very closely resembled the European specimens (the original Latin/French text and an English translation is included in the Materials and Methods Section). This suggested that we might not be dealing with a new species in Europe, but with a quite common South American species, described almost two centuries ago, whose identification was overlooked for years.

Therefore, one of us (L.E.R.) collected and photographed living specimens of the local *Photinus* species at Dique Luján (Buenos Aires, Argentina) at about 200 km (bird fly distance) from the original collection site of the *P. signaticollis*-type specimen near Maldonado in Uruguay.

**Figure 3:**
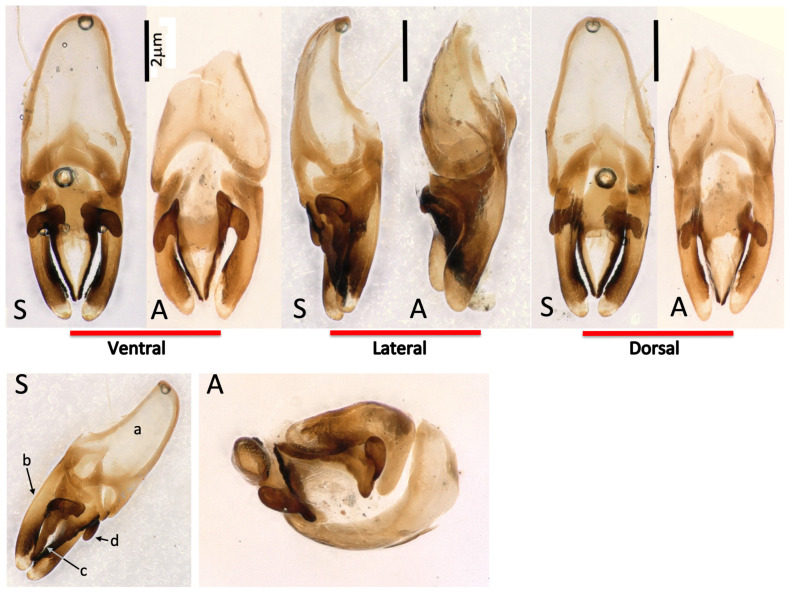
Comparison of Spanish (Extremaduran) and Argentinian *Photinus aedeagi*. Top panels: ventral, lateral and dorsal views of the *aedeagi* for a Spanish (S) and Argentinian specimen of *P*. *signaticollis* (A). Lower panels: semi-lateral (S) and bottom (A) views, facilitating the understanding of the *aedeagus* structure. *Aedeagi* are shown in the physiological head (up)–tail (down) orientation, as proposed by Dr. Lesley Ballantyne [26]; basal piece (a), lateral lobe (b), median lobe (c), dorsal basal projection (d)—aedeagal nomenclature following [13].

To show that the European and Argentinian species are identical, the species-specific male reproductive organs (*aedeagus)* were prepared from a Spanish/Extremaduran and an Argentinian specimen. As can been seen in Figure 3, the *aedeagi* are identical to each other and to the aedeagus shown in [12] of specimens from Girona. *Photinus* aedeagi are species-specific and are used for species identification [13,25].

We tried to compare the specimens shown in Figure 7 with the *P. signaticollis*-type specimen deposited in the Blanchard collection at the Museum National d’Histoire Naturelle in Paris. However, the Entomology Department of the Museum has been unable to locate the type and it may be lost. Nevertheless, the original description by Blanchard matches convincingly with the Spanish and French *Photinus* specimens, leading us to argue that *Photinus immigrans*, Zaragoza-Caballero and Viñolas, 2018, must be considered a synonym of *Photinus signaticollis* (Blanchard, 1846); this is because, according to the principle of priority (Article 23 of *The International Code of Zoological Nomenclature*, 4th edition, 1999), the valid name of a taxon is the oldest available name given to it (please note, if the type can be localized on a later date, we propose to add images in the pdf version of the online article).

### 3.7. Land Use Coverage

Our field observations seemed to indicate that these fireflies were mainly found in “open habitats”, such as cornfields, alfalfa fields (*Medicago sativa* Linneaus, 1753), permanent grasslands or well-kept garden lawns. Considering that the larva of *P. signaticollis* is a predator of earthworms, its habitat preferences will also correspond to suitable locations for its prey. Habitats, such as long-term prairies, perennial forage crops, alfalfa plots, fallow fields, riverbanks and field margins have in common that their soils remain undisturbed for enough time to provide good conditions for both *Photinus*’ larval stage and for earthworms to thrive. The large number of observations of *Photinus* also in annual crops, such as maize fields, can be explained by the patrolling and perching behavior of the males, which themselves actually come from nearby breeding grounds, such as field margins.

**Figure 4:**
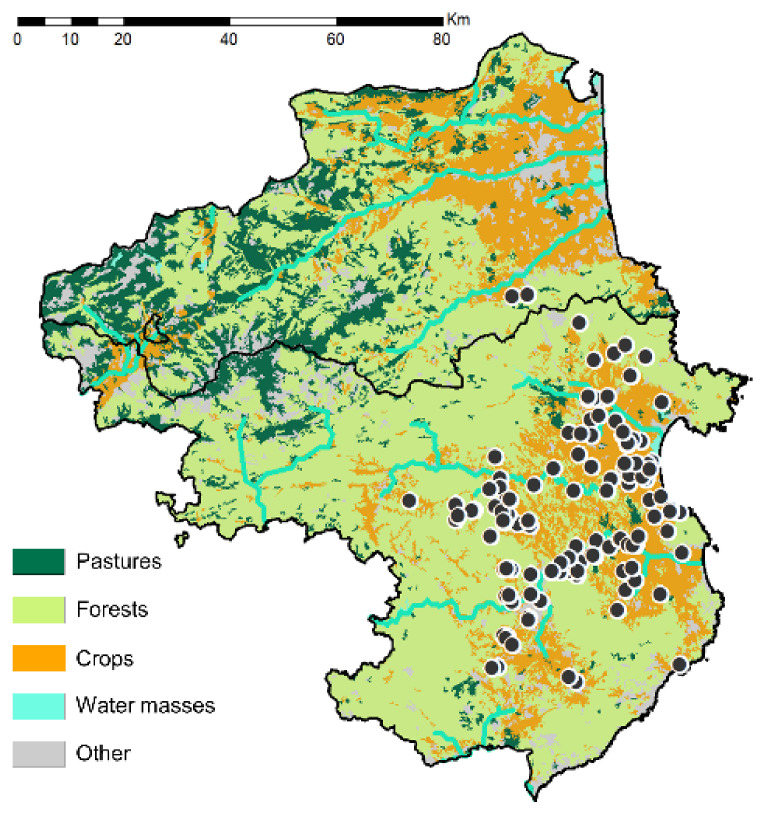
CORINE 2018 land cover map. The black “horizontal” line indicates the French–Spanish border.

The number of observations in a certain type of territory can be taken for a proxy for habitat preference of a species. To test our impression the European records of *P. signaticollis* (see Supplementary Materials Table S1) were compared with maps of land use (CORINE 2018 Land Cover), and the results are clear (Figure 4), in that the species predominantly inhabits (84% of the records) “Non-irrigated arable land” (Corine land coverage, code N°211), “Permanently irrigated arable land” (N°212), “Complex cultivation patterns (N°242), “Land principally occupied by agriculture, with significant areas of natural vegetation” (N°243) and other crops. The remaining 18 records (16%) are distributed randomly over urban areas (11%), forests (3%) and scrublands (2%).

**Figure 5:**
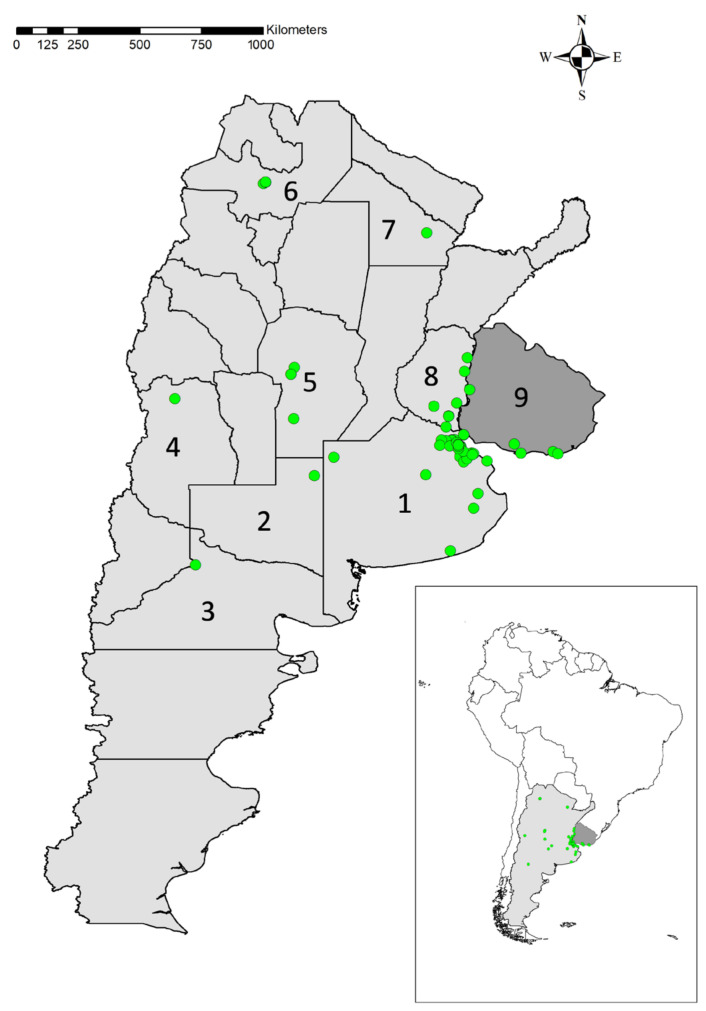
South American observations. Numbers 1 to 8 indicate the Argentinian provinces where the species has been recorded (1—Buenos Aires; 2—La Pampa; 3—Río Negro; 4—Mendoza; 5—Córdoba; 6—Salta; 7—El Chaco; 8—Entre Ríos. N° 9 indicates Uruguay (source: www.inaturalist.org; www.ecoregistros.org; www.fotosaves.com.ar (all accessed 20 October 2020)).

In South America, the same firefly is widely dispersed in parts of Argentina and Uruguay (Figure 5). According to the citizen science platforms (www.inaturalist.org; www.ecoregistros.org; www.fotosaves.com.ar (all accessed 20 October 2020), the observations have been recorded through 1600 km^2^ between 25° and 39° latitude South (the European locations range between 39° and 42° latitude North), living under equivalent climate regimes and natural and anthropogenic habitat types to the European populations. Although speculative, these also range, here, between cultivated lands, pastures, gardens, parks, coastal and dune regions. It is interesting to notice the presence of the species in the highly urbanized area of Buenos Aires D.F. (20 out of 75 observations), in parks and even in backyards in the center of the city (see Supplementary Materials Table S2).

### 3.8. Observation Phenology

**Figure 6:**
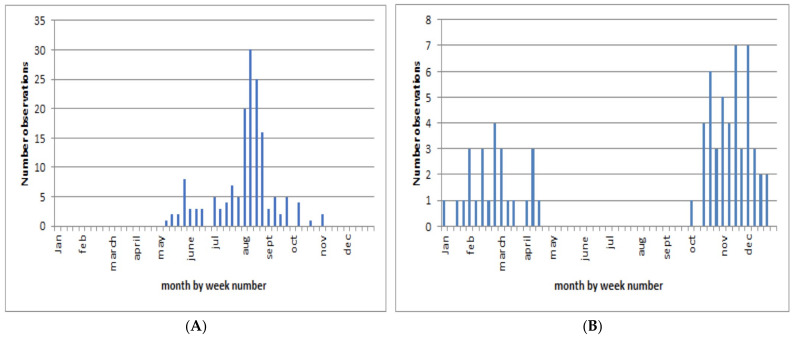
*Photinus signaticollis*—phenology. (**A**): Europe (2016–2020 observations); (**B**): Argentina and Uruguay (2014–2020 observations); 70 records from www.inaturalist.org; www.ecoregistros.org; www.fotosaves.com.ar (accessed 20 October 2020).

On the Maureillas-las-Illas site, at 150 m altitude, animals were observed from mid-June to mid-November. This extremely long flight season is in stark contrast with that of the native European species that have adult seasons lasting from 3 to 6 weeks, in general [21]. To obtain more detailed insight in the adult season, all European and South American *Photinus* observations were plotted against reporting dates (Figure 6).

Figure 6A clearly shows that the adults of this species indeed have a long flight season from May to mid-November (14 November 2020) compared with our West European species, of which (light-emitting) adults can generally be observed in France and Spain from mid-May to mid-September, but locally, these periods are often much shorter (unpublished results from OVL, R.D.C. and J.R.G.A.). The limited number of observations does not tolerate statistical analysis.

Figure 6B shows that the long adult flight season of about 6 months coincides between the South American and European populations, of course in concordance with the season inversion between both hemispheres. Moreover, according to our local contacts, this insect seems to be the only common *Photinus* species present in the Buenos Aires region.

### 3.9. Identification Help

Based on observations in the French Pyrenees and Extremadura, we found that flashing males appear quite precisely 30 min after sunset, and they keep flying for about 30 min. Both sexes produce flashes corresponding to typical species-specific bioluminescent behaviors, known from the genus *Photinus* [2,4,6]. *P. signaticollis* males perform patrol flights from a height of a few centimeters up to 5 m above the ground, while producing irregular flash trains of short flashes (<ca. 250 µs) at a flash rate of 1 flash per ca. 3.5 s, while females respond after a certain delay time (ca. 0.5–1 s) from the grassy vegetation, with a longer lasting (>ca. 500 µs) flickering single or doubled flashes in response to the male flashes (or male-imitating flashlight) (details to be published in a future paper). During this male flight period, comparatively few, very weakly (spontaneously) glowing females could also be found on the ground. Subsequently, males and females unite and, for about two hours, intense irregularly spaced flashes are emitted from the ground level.

**Figure 7:**
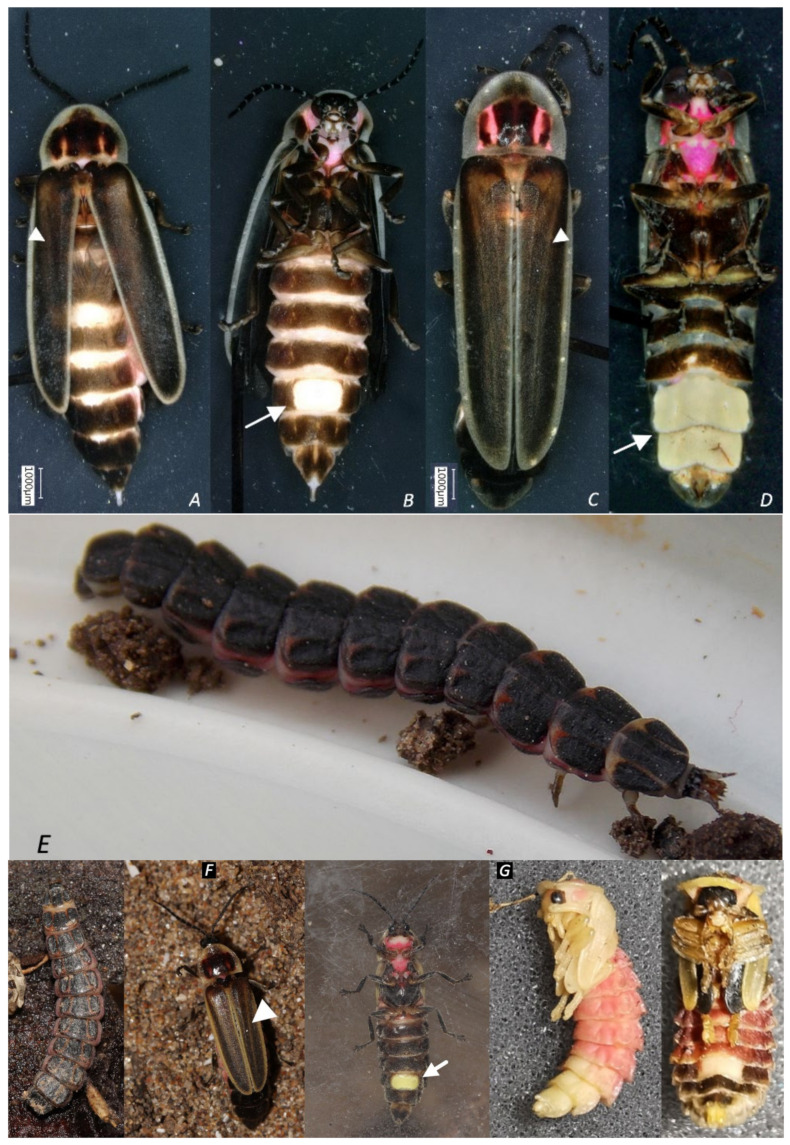
Life stages of *Photinus signaticollis*. (**A**–**D**) Adult female (France), dorsal (**A**) and ventral view (**B**); adult male (France), dorsal (**C**) and ventral view (**D**). Arrows indicate the lanterns; arrowheads show the vittae (**E**,**F**). Larvae (Argentina, photo credits L.E.R.). (**F**) Larva (left panel) and the female (**F**), middle and right panel) that hatched from it. Arrow indicates the lantern. Arrowhead shows a vitta. (**G**) Pupae (Spain) (left—male; right—female).

To allow citizens and scientists to easily recognize the arrival of these particular animals and help to follow their dispersal in the future, the known life stadia are depicted in Figure 7.

The elongated form of the animals, the red marks and black square on the pronotum as well the slightly light brown line pattern on the dark brown elytra (vittae) (Figure 7A,C,F (middle panel, arrowheads) are completely unique and absent from European fireflies and glow-worms and permit easy recognition (for more detailed field recognition characteristics, see Materials and Methods Section).

Males have a lantern that covers two segments (Figure 7D, arrow), whereas the female’s lantern is limited to a small part of a single segment (Figure 7B, arrow). As can be seen, and unlike all native European species, both male and female are fully winged. While finalizing the current study, the larval stage of this firefly has also been reported in Catalonia [27]. Local Argentinian naturalists told us that the morphotype of *P. signaticollis* is the only *Photinus* firefly commonly encountered in the habitats we prospected, and a local *Photinus* larva was found and raised by L.R. (Figure 7F). The animal pupated and resulted in a female *P. signaticollis* (Figure 7F). We were recently also able to make females from Maureillas-las-Illas (France, September 2021) lay eggs and breed them through the different larval stages (R.D.C., work in progress).

Pupae (Figure 7G) were found on a known *Photinus* location in Girona (Spain) and resemble the classical *Photinus*-type. As no other members of the genus are currently known to be present in Spain, there is a high likelihood that these specimens are indeed *P. signaticollis* pupae.

## 4. Discussion

Our data clearly demonstrate that we are dealing with an introduced species that seems to be expansive, if not even invasive, as it dispersed very rapidly throughout Catalonia and recently entered France. The most likely hypothesis about the introduction of this exotic firefly is that the larvae or pupae of *P. signaticollis* have been moved into Europe by indirect and passive transport of their larvae/pupae through gardening activities by importing plants with soil from South America.

The fact that females have well-developed wings, and probably fly well, in contrast to all autochthonous European Lampyrids, could be one of the reasons that this species is so successful in colonizing new sites. In European Lampyrids, dispersal is only possible via the (female) larval stage, since adult females are rather short-lived, flightless and, as such, nearly immobile.

Assuming that the whole country’s surface can be colonized, and with a 10 km/year dispersal, a rough calculation shows that the 543,940 km² of metropolitan France could be covered in about 40 years, and smaller Spain in even less time. This is, of course, without considering facilitation of spreading by winds or human intervention that could largely augment dispersal.

Our results show that *P. signaticollis* shares characteristics (spread by annual dispersal, high settlement capacity, high local abundance, etc.) with invasive species, such as *Harmonia axyridis* (Pallas, 1773) (Coleoptera: Coccinellidae), a ladybug that rapidly colonized different countries with dispersal rates estimated between 100 and 500 km/year [28]. However, our results for *P. signaticollis* show much lower dispersal speeds (6–10 km/year) than *H. axyridis*, probably due to a slower flight capacity that reduces dispersal.

This smaller dispersal capacity, and its actually quite localized distribution, can hopefully be used to develop population control programs, before this species colonizes new European areas.

Deliberate dispersal (personal experience: R.D.C., J.R.G.A., M.K. [29]) will be another important issue to consider, especially in the future, as fireflies are attractive to people to own as a display feature in their gardens. An example of human introduction of flashing fireflies is the case of *Luciola italica* (Linnaeus, 1767) in the Parc Bourget near Lausanne (Switzerland) [30]. With this particularly attractive *Photinus* species, this could represent an important reason for quick (and potentially harmful) dispersal.

The fact that the population in Extremadura seems to be confined to a unique location (or, at least, until now, as we were unable to find any nearby populations) urges more future research in order to understand this situation. This is especially the case while considering the long time period during which the owner of the site claims to have witnessed this population.

We cannot yet confirm efficient female flight for the Extremaduran population (persons observing R.D.C.). Five females were tested in several attempts on each individual to incite them to fly. None did fly, except sometimes a hopping flight-jump of about 3–4 cm.

This potential loss of flight capacity of Extremaduran females may be a way to explain their apparent non-dispersal.

Regarding the flight capacity of the species, the study of the Catalan population led us to hypothesize a sequential reproductive and distribution strategy, as otherwise dispersal is impossible to explain. In the beginning of the adult season, young females may be full of eggs and too heavy for long flights, and thus nurture the local population. However, late-season females, having lost weight by laying most eggs, are, on the contrary, able to fly and can thus colonize new territories at the end of the reproductive season [31].

A factor that certainly also aids dispersal is the extremely long adult flight seasons found in our phenology analyses both in Europe and Argentina. Two hypotheses may explain this. Either, throughout the whole season, pupae constantly give rise to new flashing adults, or we are dealing with a single appearance of very long-living adults, a possibility that is perhaps supported by our difficulty in finding larvae. Long adult seasons with multiple seasonal waves or peaks are known in other species, such as *Photinus pyralis* (Linnaeus, 1758) [2]. However, in the second hypothesis, the fireflies would need resources to ensure longevity. This would heavily contrast with the native fireflies and glow-worms, where adults do not consume food—at best, they consume a bit of liquid [21]. Adult *Photinus* fireflies in the Americas are known to consume at least nectar and pollen from flowers and lick saps from plants, possibly even acquiring plant toxins to incorporate them into their own chemical defenses [2,4,32]. This, together with intricate reproductive strategies—where females get beside spermatophores also nuptial gifts from their males, which they can either invest in producing more offspring or in longevity [4,33,34]—might explain an extended adult lifespan, in contrast to the native European glow-worm and firefly species that usually live from one week to about 3 weeks maximum [35,36,37]. *Photinus* females in contrast to our European Lampyrids [21,36,37] can mate multiple times [38] and this could suggest that females, although having already fertilized all their eggs, continue looking for a good supply of nutritious spermatophores/nuptial gifts to supplement their remaining larval reserves and, in this way, benefit from a longer adult life span.

Scanning electron microscopy and numerical Keyence microscope analysis of four male and two female specimens systematically showed that *P. signaticollis* was covered with soil (not shown), probably betraying the daytime hide (and potentially hunting grounds) of these animals—underground. These *Photinus* females may reside during the daytime in burrows, as seen in other species [38,39]. Often, males, especially during long mating sessions, in species where males transmit nuptial gifts in the form of spermatophores [4,33,34], are dragged into the female underground hiding places during the mating ritual [38,39], which may also explain the soil found on the specimens.

Due to this possible underground life, citizen scientists will be asked to pay careful attention to adults, larvae and pupae while working in their gardens and crops.

Through the rediscovery of *P. signaticollis* described here, we hope that this type of work will increase the interest in studying beetles and emphasize the importance of scientific collections in natural history museums. This work again underlines the importance of citizen science and photo-sharing platforms that can provide a wealth of data for the detection, monitoring and study of native species as well as exotic and invasive species [8,10].

Our work in progress includes performing DNA analyses on specimens caught in France, Spain and Argentina. Aedagus analysis is generally considered as strong evidence for species identities. DNA sequencing will provide complementary data on the relations between the different populations and elucidate where the European populations came from.

GDL has been receiving further unverified reports of flashing and flying fireflies from other parts of Spain. Additionally, in the Perpignan region, some old and unclear reports of “a flickering firefly in flight” exist [40]. The question that now remains is whether this species is already present elsewhere in Spain and France. If this is the case, we pose the following question: why did the species start, all of a sudden, and very efficiently, establishing itself in new regions? Alternatively: are we simply dealing with new introductions of ultra-fit specimens from the Americas?

Little behavioral information is available for most described *Photinus* and related genera (i.e., the genera *Lucidota*, *Phosphaenus*, *Phosphaenopterus*). Up till now, all larvae that have been studied seem to be specialist oligochaete earthworms predators (personal observations: R.D.C.; personal communications: Lynn Faust, Sara Lewis [4]).

Additionally, the only two native Spanish and French firefly species belonging to the same tribe (Photinini) are known (*Phosphaenus hemipterus* (Geoffroy in Fourcroy, 1785)) or suspected (*Phosphaenopterus metzneri* Schaufuss, 1870) earthworm predators [41]. Note that the European lesser glow-worm, *P. hemipterus*, was accidentally introduced in Canada, probably with agricultural and horticultural products, and continues to eat its European prey: the previously also introduced earthworm, *Lumbricus terrestris* Linnaeus, 1758 [42]. So, it seems most likely that also the larval *P. signaticollis* will feed on oligochaetes, a behavior that differs from almost all other native French and Spanish Lampyrids, whose larvae prey on snails and slugs. The introduction of a new earthworm predator may have unpredictable consequences for the native oligochaete fauna, which in France is already under attack by several species of invasive platyhelminths [43,44]. Hopefully, these newcomer fireflies do not also consume slugs and snails, as in this case, they would enter in direct competition with (or even worse, eat) most of our native glow-worms and fireflies. This could have occurred if not a *Photinus* but a *Photuris* species, known to eat other fireflies, had invaded our pastures [3], as we know that European Lampyrinae typically contain defensive lucibufagins, which are wanted by this American genus (*Photuris* eat male *Lampyris noctiluca* (Linneaus, 1758); personal observations: R.D.C. [45]). Our native glow-worms and fireflies suffer heavily already under light pollution [46,47,48], and, as many other insects, from the overly intensive mowing of roadsides. Additionally, the widespread use of insecticides and limacides seriously hampers them, and they are thus not waiting for yet another threat.

Although the *Photinus* fireflies give magnificent light displays during summer evenings, normally never seen in Europe, the probable risk they represent for the native fauna should drive us to try and remove these animals from our countryside and to avoid both accidental and deliberate introductions. Therefore, we think it is advisable to design actions to specifically control the populations of *P. signaticollis* in Spain and France. The bioluminescence emission color (575 nm and 595 nm) and the flash rhythm of *P. signaticollis* (male patrol flash: ca. 1 flash/3.5 s; paper in preparation) is quite different from that of the native glow-worms that show a continuous courtship with a lime green glow (around 550–555 nm) [49,50,51]. Only *L. lusitanica* flashes with similar light colors (570 nm) but their rhythms seem different, with males showing a faster flash pattern of ca. 2 flashes/s [19]. Therefore, it may still be possible to specifically minimize the proliferation of the exotic *Photinus*, before they invade too many places in France and Spain.

For all the above-mentioned reasons, we are convinced that it is of utmost importance to quickly design and fund programs to study the biology of this exotic species and to monitor its rapid spread throughout Europe.

## Data Availability

Provided as Supplementary Materials.

## Supplementary Materials

The following supporting information can be downloaded at: https://www.mdpi.com/article/10.3390/insects13020148/s1, Table S1: Raw data from Spanish and French observations analyzed in this article, Table S2: Raw data from Argentinian and Uruguayan observations used in this article, Supplementary Data S1: providing the following: online search strategy, determination features, and a list of South American observers.
