# Supplementary material for: Quick Spreading of Populations of an Exotic Firefly throughout Spain and Their Recent Arrival in the French Pyrenees
Source: Insects. 2022 Jan 29;13(2):148. doi: 10.3390/insects13020148 (PMC8880130; doi:10.3390/insects13020148)
Supplement: Supplementary file 1 [file insects-13-00148-s001.zip › insects-sup-1538234/suppl mat/Sup.Data S1-Quick spreading.pdf]

Supplementary Material: Quick spreading of populations of an exotic firefly throughout Spain and their recent arrival in the French Pyrenees

Online Search strategy

Photographs of *P. immigrans/P. signaticollis* lookalikes were searched on the internet on various platforms. First, we searched google images with the search terms "*Photinus*" & "Uruguay" and "*Photinus*" & "Argentina" and "luciérnaga" (firefly in Spanish) & "Argentina" and "luciérnaga" & "Uruguay", which immediately led us to material on EcoRegistros.org and www.fotosaves.com.ar .

In order to decide whether we were dealing with *P. immigrans/P. signaticollis*, the discriminatory features stated in M&M were applied.

EcoRegistros was further searched for photographic specimens by JRGA and RDC by scouring the platform on the search terms "Lampyridae", "luciérnaga" (firefly in Spanish) and "Photinus". RDC searched the online platform iNaturalist.org for specimens throughout South America for "Photinus", "Lampyridae" and "Lampyrinae".

At a later stage we also searched online for "*Photinus signaticollis"* but that did not yield any images or photos.

MALES

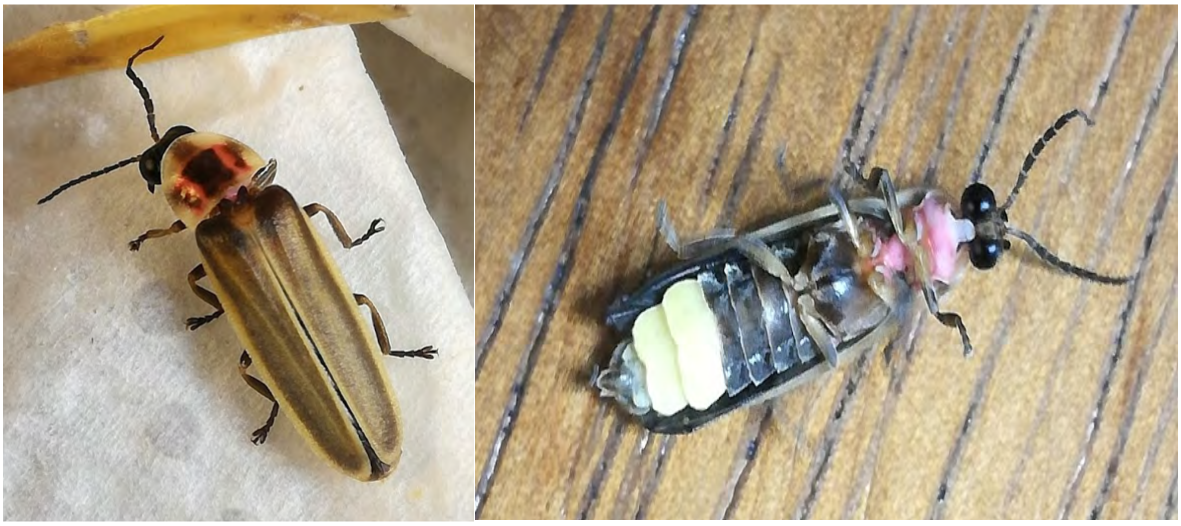

Male with typical pronotum coloration, slender; type with tapering elytra. More pale than typical colour, shoulder stripe (vitta) present but vage and blurry, scutellum quite pale overall (usually darker with paler margin)

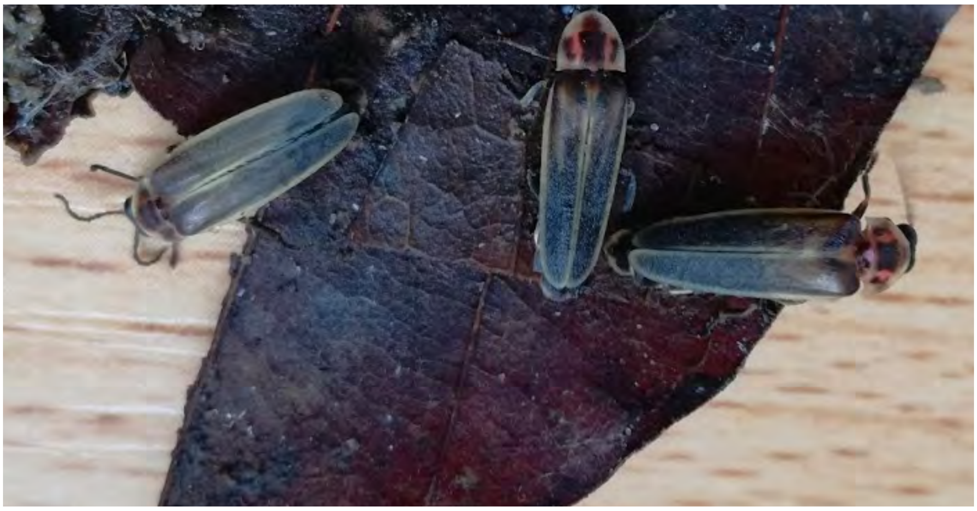

Figure S1. Three males with less clear or incomplete shoulder stripes (vittae) and darkened elytral tips except of the pale margins. Note the typical tapering body shape of all; broadest at shoulders

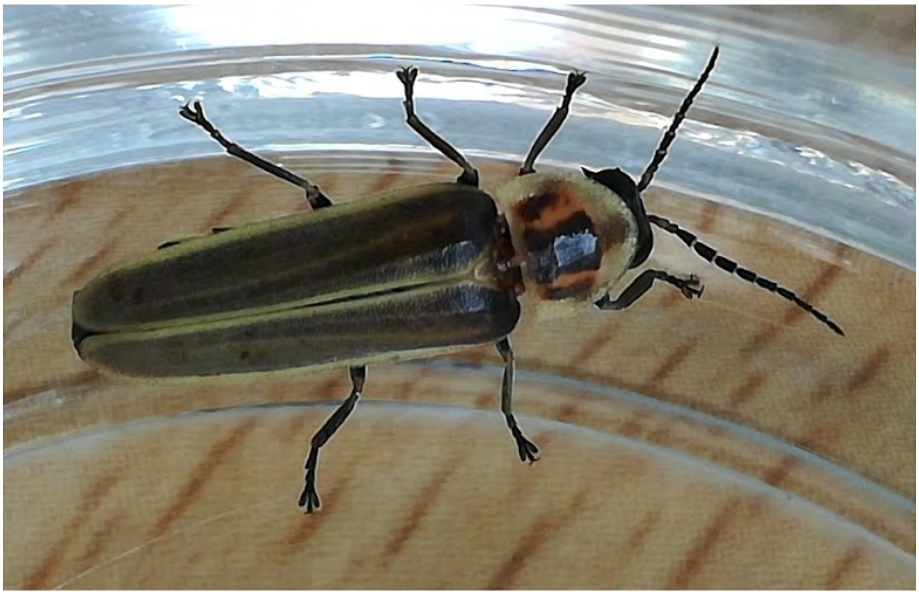

Figure S2. Typical male, with shoulder vittae; pink line pronotum somewhat broadened anteriorly.

FEMALES

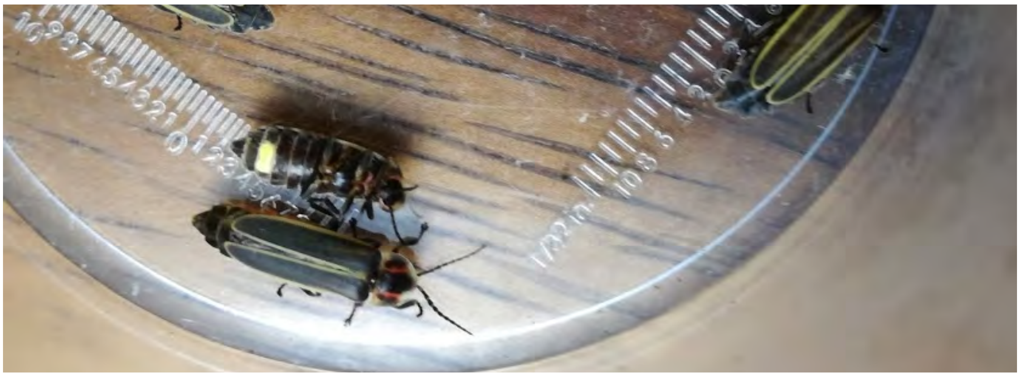

Figure S3. Four females, one with ventral view, 2 up with very clear pale shoulder stripes (vittae), one below with much less distinct vittae and somewhat darker elytra; all show typical pronotum colouration

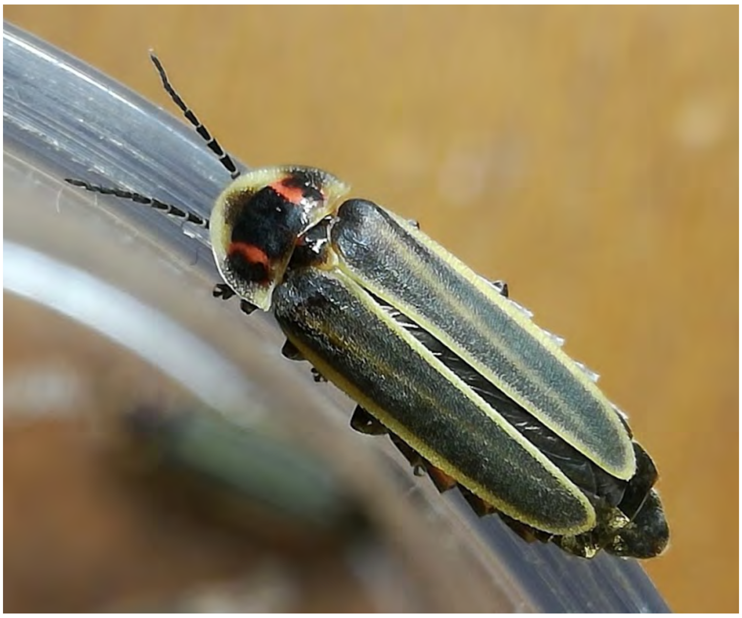

Figure S4. Female with pronotal pink markings clearly present with a more marked central thickening on the line shape.

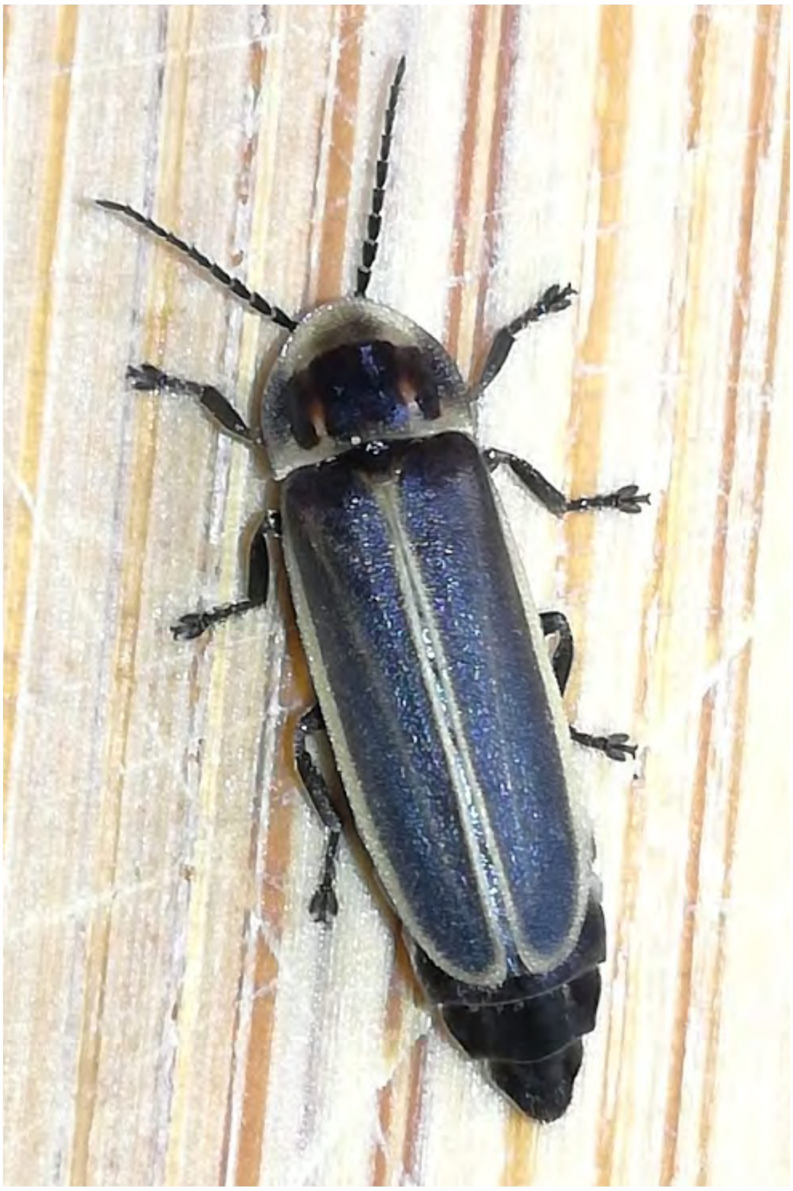

Figure S5. Female with vague almost absent shoulder vitttae on quite dark elytra, and quite dark pronotum with very unclear pink pronotum markings.

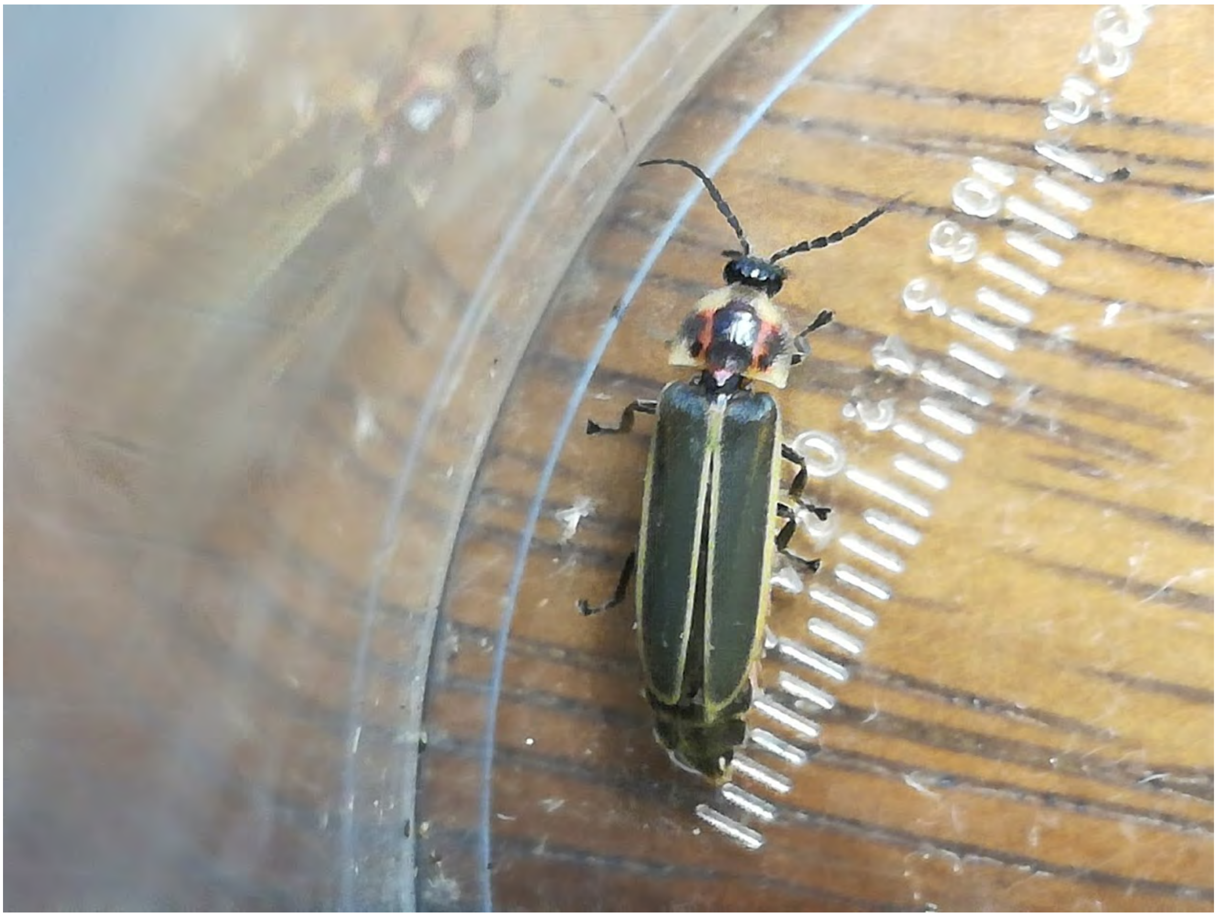

Figure S6. Female with typical pronotum but very dark almost bmlack instead of smoke grey elytra with only very vague start of shoulder stripes, very distinct pale elytral margins.

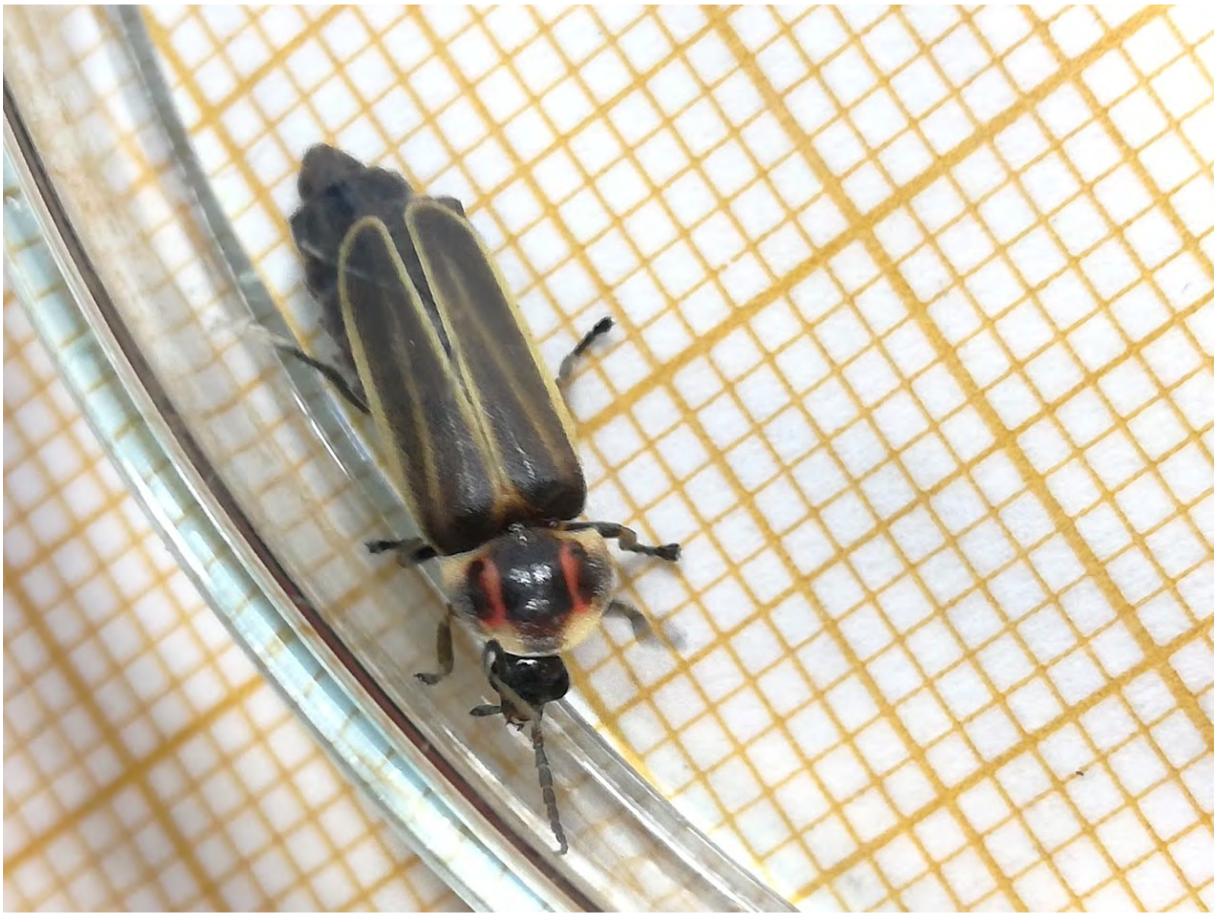

Figure S7. Typical female with with vittae

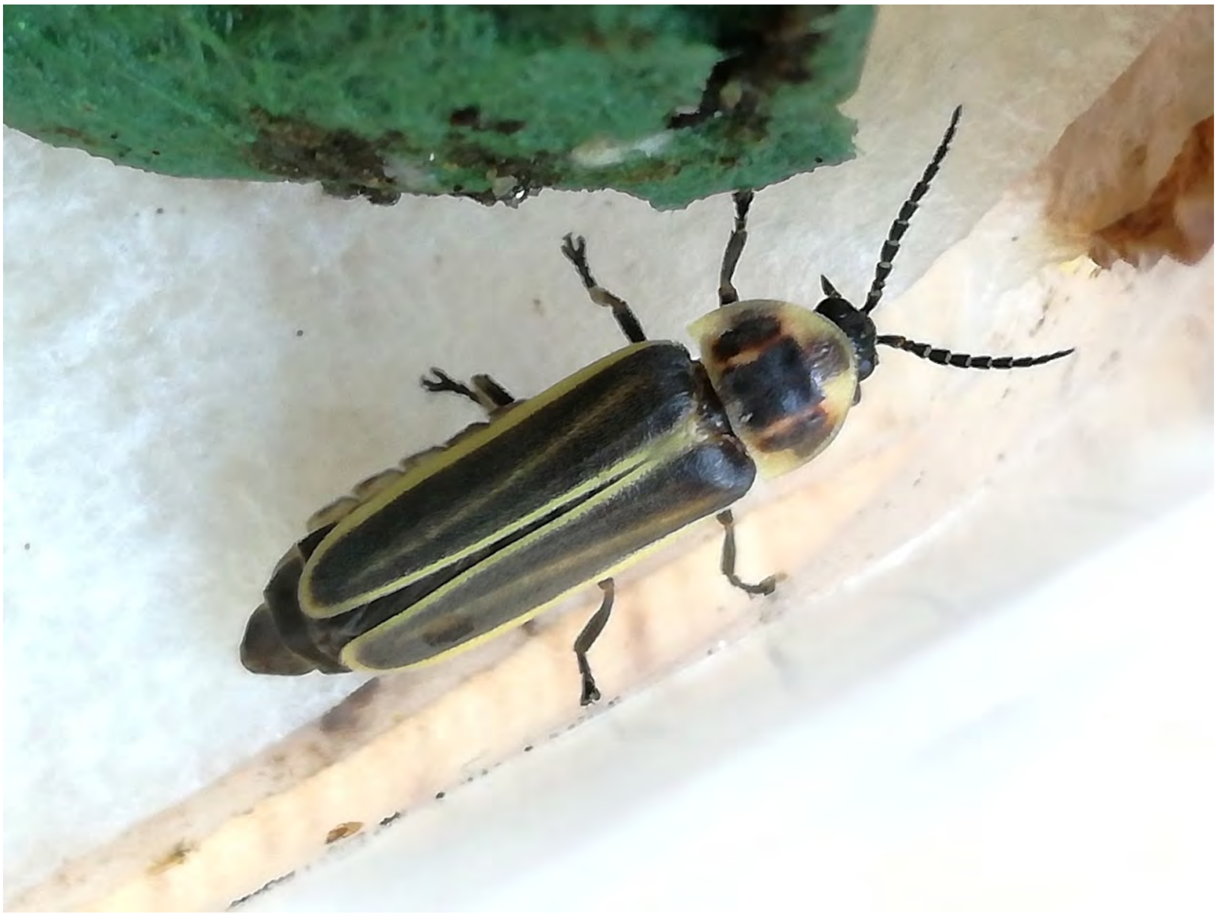

Figure S8. Female with typical coloration with shoulder vittae, but very faint and thin pink pronotal line markings

COUPLES

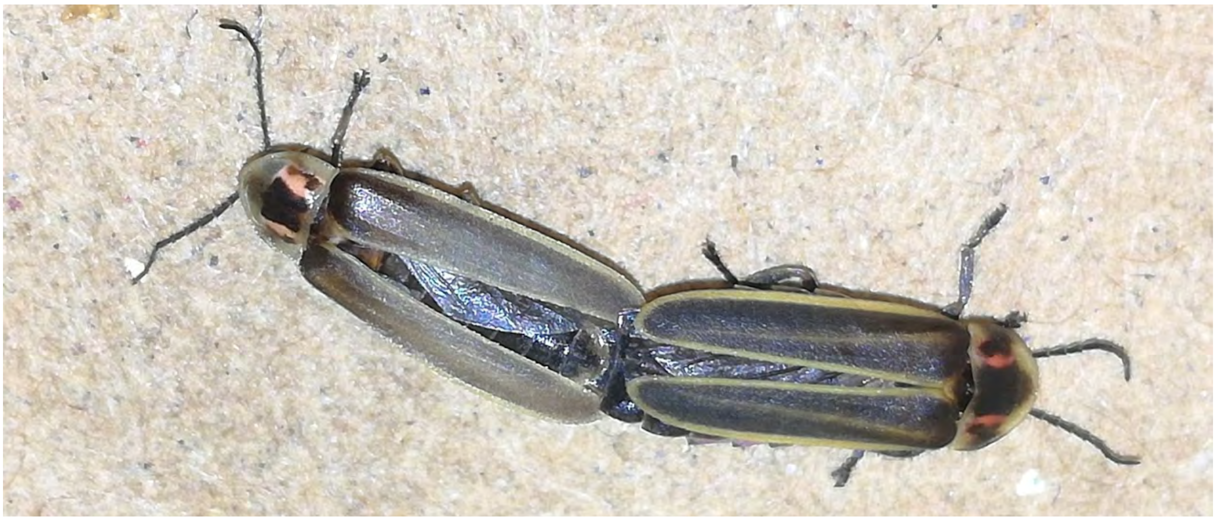

Figure S9. Male left, female right. Male more greyish elytra, without clear shoulder vittae and less yellowish (more greyish) elytral margins.

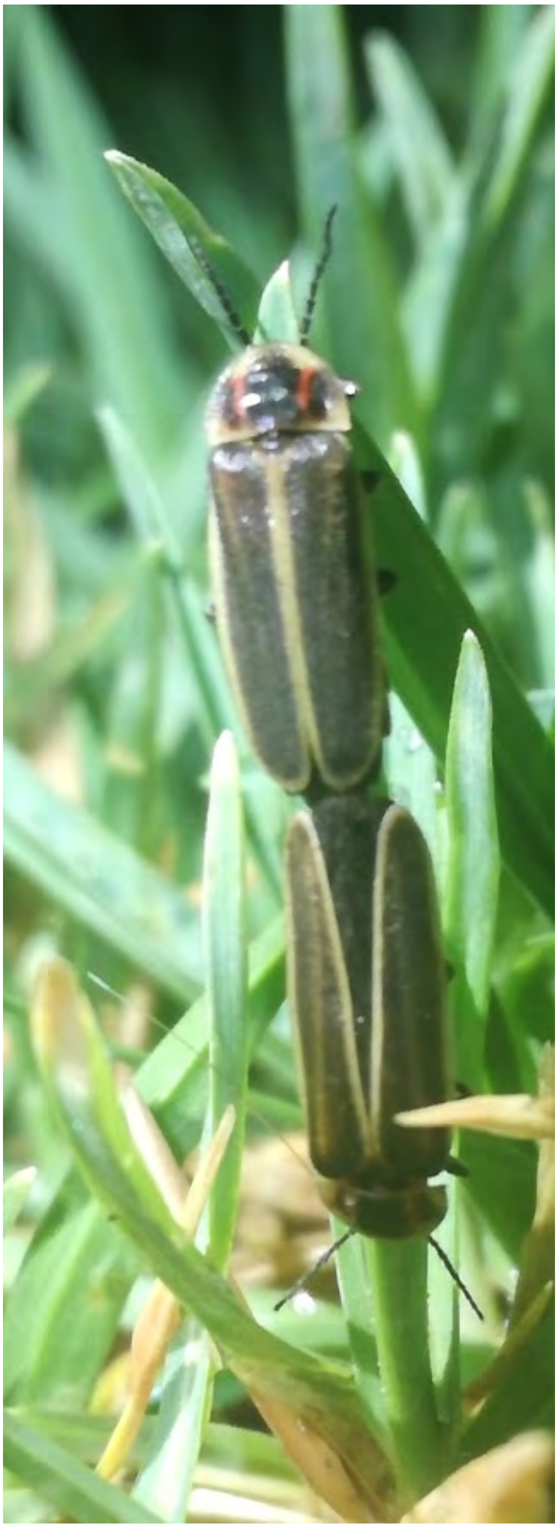

Figure S9. Typical 'Photinus' mating positions; first they start with male on female (position 1; ref. see Lynn Faust), to position 2 tail-to-tail (this photo); female up, male below. In *P. immigrans* females typically are always positioned head up on grass leafs; male clear vittae along whole elytra, female with shoulder vittae less clear from halfway elytra.

List of observers South America

Observers from Argentina in www.ecoregistros.org: Marcelo Zanotti, Diego Alfonso Rosa, Eduardo Beltrocco, Eduardo Nadal, Gustavo Peretti, Gustavo Puente, Jorge Iriberri, Lucas Rubio (also co-author), Luis Carlos De Volder, Mariano Kildegaard.

Observers from Argentina in www.fotosaves.com.ar: Alec Earnshaw

Observers from Argentina in www.inaturalist.org: "Emiss", "RAP", Abril Bastons, Candela Escudero, Diego Carús, Diego Emmanuel Oscar Diego Ese Olivera, Eduardo Luis Beltrocco, Emmanuel Zufiaurre, Ezeq DeRegibus, Federico "buddyfede", Gabriel Albelda, Gerónimo Martín Alonso, Gonzalo Roget, Humberto Debat, Inecaatro, Isadora, Isis Ibáñez, jfps, Juan José Bonanno, Juan Manuel Atencio, Leonel Roget, Lorena Zapata, Lucas Rubio (co-author), Luis Martinez Villa, Margarita Cervio, María Belén Dri, Martin Arregui, Michelle Delaloye, Nico Chimento, Pablo Bombín, Raúl Orencio, Ricardo Ernesto Doumecq Milieu, Roberto Battaglia, Roberto Guller, Rodrigo Carrera, Seba, sebinio28, Sebastián Fornés, Sol Quipildor, Verónica André

Observers from Uruguay in www.inaturalist.org: Bert “bert_in_the_skirt”, Francisco Liguori, Miguel Latronico,Pablo Balduvino, Santiago Mailhos

List of online data of observations in Argentina and Uruguay used in this paper:

https://www.inaturalist.org/observations/9092448
https://www.inaturalist.org/observations/60103447
https://www.inaturalist.org/observations/53426875
https://www.inaturalist.org/observations/36066055
https://www.inaturalist.org/observations/41019842
https://www.inaturalist.org/observations/35325161
https://www.inaturalist.org/observations/20787769
https://www.inaturalist.org/observations/18805968
https://www.inaturalist.org/observations/36166849
http://www.ecoregistros.org/site/imagen.php?id=370411
http://www.ecoregistros.org/site/registro.php?id=1128786
http://www.ecoregistros.org/site/imagen.php?id=299720
http://www.ecoregistros.org/site/imagen.php?id=198108
http://www.ecoregistros.org/site/imagen.php?id=361344
http://www.ecoregistros.org/site/imagen.php?id=320878%20
https://www.inaturalist.org/observations/18316136
http://www.ecoregistros.org/site/imagen.php?id=187582
http://www.ecoregistros.org/site/imagen.php?id=245055
http://www.ecoregistros.org/site/imagen.php?id=310238
http://www.ecoregistros.org/site/imagen.php?id=327273
http://www.ecoregistros.org/site/imagen.php?id=318191
http://www.ecoregistros.org/site/registro.php?id=905121
http://www.ecoregistros.org/site/registro.php?id=1209748
https://www.inaturalist.org/observations/38162436
https://www.inaturalist.org/observations/9117905
https://www.inaturalist.org/observations/35608451
https://www.inaturalist.org/observations/19868490 https://www.inaturalist.org/observations/18339097

https://www.inaturalist.org/observations/36481364
https://www.inaturalist.org/observations/36825861
https://www.inaturalist.org/observations/47019535
https://www.inaturalist.org/observations/18558716
https://www.inaturalist.org/observations/50987617
https://www.inaturalist.org/observations/63602039
https://www.inaturalist.org/observations/36879280
https://www.inaturalist.org/observations/63592704
https://www.inaturalist.org/observations/19792046
https://www.inaturalist.org/observations/62719275
https://www.fotosaves.com.ar/FotosInsectos/FotosInsectos.html
https://www.inaturalist.org/observations/65631338
https://www.inaturalist.org/observations/65584286
https://www.inaturalist.org/observations/65269471
https://www.inaturalist.org/observations/65241011
https://www.inaturalist.org/observations/65008008
https://www.inaturalist.org/observations/64859026
https://www.inaturalist.org/observations/64780035
https://www.inaturalist.org/observations/64556725
https://www.inaturalist.org/observations/64260800
https://www.inaturalist.org/observations/66230021
https://www.inaturalist.org/observations/66580476
https://www.inaturalist.org/observations/66718299
https://www.inaturalist.org/observations/67070783
https://www.inaturalist.org/observations/68934936
https://www.inaturalist.org/observations/70739419
https://www.inaturalist.org/observations/70147416
https://www.inaturalist.org/observations/70121583
https://www.inaturalist.org/observations/70059457
https://www.inaturalist.org/observations/69646262
https://www.inaturalist.org/observations/69534366
https://www.inaturalist.org/observations/75447143
https://www.inaturalist.org/observations/75359106
https://www.inaturalist.org/observations/74041936
https://www.inaturalist.org/observations/65793673
https://www.inaturalist.org/observations/65585468
https://www.inaturalist.org/observations/63592695
https://www.inaturalist.org/observations/63480745
https://www.inaturalist.org/observations/63279869
https://www.inaturalist.org/observations/59144904
https://www.inaturalist.org/observations/56677379
https://www.inaturalist.org/observations/53558355
https://www.inaturalist.org/observations/39798626
https://www.inaturalist.org/observations/42035277
https://www.inaturalist.org/observations/38488652
https://www.inaturalist.org/observations/36122001
https://www.inaturalist.org/observations/25925424
https://www.inaturalist.org/observations/25264292
https://www.inaturalist.org/observations/22166563
